# Cellular superresolved imaging of multiple markers using temporally flickering nanoparticles

**DOI:** 10.1038/srep10965

**Published:** 2015-05-28

**Authors:** Tali Ilovitsh, Yossef Danan, Rinat Meir, Amihai Meiri, Zeev Zalevsky

**Affiliations:** 1 Faculty of Engineering, Bar Ilan University, Ramat-Gan 5290002, Israel; 2 The Bar-Ilan Institute of Nanotechnology & Advanced Materials, Bar Ilan University, Ramat-Gan 5290002, Israel; 3 Department of Electrical and Computer Engineering, University of Utah, Salt Lake City, Utah, USA

## Abstract

In this paper we present a technique aimed for simultaneous detection of multiple types of gold nanoparticles (GNPs) within a biological sample, using lock-in detection. We image the sample using a number of modulated laser beams that correspond to the number of GNP species that label a given sample. The final image where the GNPs are spatially separated is obtained computationally. The proposed method enables the simultaneous superresolved imaging of different areas of interest within biological sample and also the spatial separation of GNPs at sub-diffraction distances, making it a useful tool in the study of intracellular trafficking pathways in living cells.

Molecular imaging of cells is an important tool for the investigation of biological systems. The ability to trace and track different cells or different components within a cell has an important role in understanding cellular trafficking pathways, identifying receptor expression and facilitating the understanding of cellular signaling pathways which can lead to the design of effective therapies for medical applications^1^,^2^,^3^,^4^,^5^,^6^,^7^. Therefore, a method for identifying the binding of labeling markers to a cell and the ability to trace it is of great importance. One common method for labeling is to use fluorescent dyes and fluorescent proteins (FPs) as biomarkers^8^,^9^,^10^,^11^. However, these markers are photo toxic to living cells and their photochemical activity is destroyed after multiple cycles of switching on and off and in addition, their use is limited to visible wavelengths^12^,^13^,^14^,^15^,^16^. GNPs provide an alternative choice for labeling as they are nontoxic, have a long lasting activity, are inexpensive and easy to produce and have spectral absorption/reflectance peak suitable for a variety of wavelengths^17^,^18^,^19^.

GNPs exhibit localized surface plasmon resonance (SPR), which is manifested by enhanced absorption and scattering at a specific optical frequency when are under optical illumination that matches this resonant wavelength^20^. The peak resonance wavelength of the GNPs is governed by their shape, size and the refractive index of the environment^21^,^22^,^23^,^24^. The most basic GNP shapes include spheres, which have a peak resonance around *532* *nm* (depending on their exact dimensions), and rods which usually have two resonance peaks; one due to the transverse oscillation of the electrons (which may be around *520* *nm*) and the other due to the longitudinal plasmon resonance at longer wavelengths that depends on the aspect ratio of the nanorod. The larger the aspect ratio is, the wavelength of the second resonance will become longer. Recently, the use of gold nanoparticles (GNPs) as biomarkers has been demonstrated^25^,^26^,^27^,^28^, however, the ability to simultaneously trace different types of GNPs that are site-specifically labeling tissues, cells or areas within a cell is a difficult and more complicated task. One reported study uses a hyperspectral darkfield microspectroscopy system that was developed in order to record the scattering spectra of cells labeled with molecular tags that scatter strongly in distinct spectral windows. This system operated using specific types of GNPs and requires a custom designed system^29^.

Another limitation stems from the diffraction limit. When imaged, each GNP becomes a point spread function (PSF) due to the diffraction limit set by the Rayleigh criterion^30^. As a consequence, areas with high GNPs concentration will appear as large spots. This can be a limitation for applications that require high precision for example, the defining of tumors for their guided surgical resection without damaging normal tissues or vital areas. In addition, this is also a limitation for localization microscopy techniques that require images with isolated PSFs that don't overlap^31^.

In this paper we use the temporally sequenced labeling (TSL) technique^32^ for the simultaneous detection of multiple GNPs labeling a sample, and their separation at sub-diffraction distances. The proposed technique is simple and requires almost no additional components, therefore is such that existing setups could be altered to incorporate the proposed approach without a necessity for major modifications.

## Theoretical background

The TSL technique uses lock-in detection to obtain higher contrast in single wavelength imaging of GNPs. Here we use *M* simultaneously modulated laser beams with wavelengths that match the GNPs plasmon resonance. Each laser is modulated with a known and different temporal frequency of *ν*_*0i*_, where *i* *=* *1…M* is the index of the modulated laser. The light scattered from the sample is captured as a temporal sequence of intensity images, at a frame rate that is more than double the flickering rate (Nyquist rate)^33^. The intensity of each image is proportional to the sum of the temporally sampled modulated signals and some additive noise:
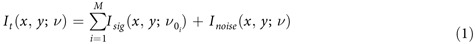
where *I*_*t*_ is the image intensity, *I*_*sig*_ is the signal intensity without noise, *I*_*noise*_ is the noise intensity. A temporal spectral analysis is performed on the received sequence of spatial images. Since the information obtained from each type of GNP lies in a specific spectral component that corresponds to its modulation frequency, it can be extracted computationally. The post processing can be performed using any numerical package (e.g. MATLAB, MathWorks, Natick, MA, USA) for each modulation frequency separately. The reconstructed image is the average sum of the set of images convolved with the corresponding modulation frequency:
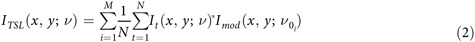
Where *I*_*mod*_ is the intensity of the modulation signal, N is the number of the images that were captured and *t* *=* *1…N* is the index of each image. The result is an image with distinct separation between the elements that have different modulation frequencies, even in cases were the two GNPs are at such sub-diffraction distance that in conventional method appear as a single spot. In addition, the wide spread spectrum noise is significantly attenuated in respect to the signal as the signal is correlated to a specific frequency, thus significantly increasing the SNR.

### Simulations

To simulate the proposed method, we have generated a set of artificial data including random emitters at each set. In these simulations the model was of a sample that contains two types of GNPs with emission peak at wavelengths of *λ*_*1*_ *=* *532* *nm* and *λ*_2_ *=* *785* *nm*. The sample was illuminated simultaneously using two temporally modulated lasers at wavelengths that match the GNPs emission peaks. The frame rate was of *100 frames/s*, *ν*_*01*_ *=* *13* *Hz and ν*_*02*_ *=* *25* *Hz* to match those of our experimental setup. Shot noise was added as a Poisson process with an expected value which corresponds to the noiseless pixel values and a standard deviation (STD) that equals the square root of the value of each pixel. Background noise was introduced by adding a sample from a Poisson distribution random variable with variance *N*_*b*_ (assumed constant across the field of view)^34^.

**Figure 1:**
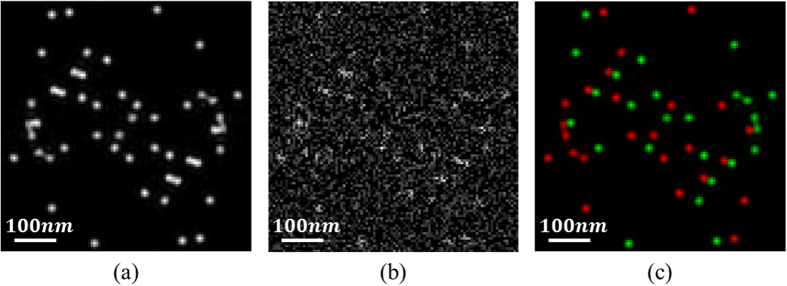
Simulation results. (**a**) The simulated sample with random diffraction limited GNPs. (**b**) A single image from the set of generated images with added noise, corresponding to SNR of -10dB. (**c**) The reconstructed image after applying the TSL technique for each frequency and summing the two images. The two types of GNPs are marked in red and green. The SNR of the reconstructed image is 30 dB.

A sequence of time dependent images was generated and analyzed. Using the a priori knowledge of the temporal modulation frequencies, the set of images were convolved with each of the modulation signal frequencies separately, followed by time averaging. The result is two different images where each image contains a single type of GNP. The complete image of the sample is a simple sum of these two. Figure 1(a) is the simulated sample with random diffraction limited spots originating from scattering from the two types of GNPs. Noise was added to the image, corresponding to SNR of −10 dB (Fig. 1(b)). The SNR was calculated according to:
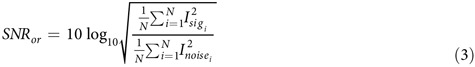


The TSL was applied on each of the two frequencies, which yielded two different images. For visualization, the GNPs in each image were colored differently (in green and red, corresponding to laser wavelengths). The reconstructed image is a simple sum of the two (Fig. 1(c)). The calculated SNR is 30 dB. The proposed technique enables the extraction of the GNPs signal even at poor SNR of −50 dB. In addition, large diameter spots in the original image (Fig. 1(a)), are usually a result of close proximity GNPs. After the processing, individual GNPs become distinguishable within the spots, as can be seen in Fig. 1(c). The method's ability to detect overlapping types of GNPs, at distances much smaller than the diffraction limit, makes it attractive for super resolving localization microscopy techniques.

## Materials and methods

### Synthesis and conjugation of spherical GNPs

Spherical GNPs (shape *20* *nm*, peak at wavelength of *532* *nm*) were prepared using sodium citrate according to the known methodology described by Enustun and Turkevich^35^. 0.414 mL of 1.4 M HAuCl_4_ solution in *200* *mL* water was added to a *250* *mL* single-neck round bottom flask and stirred in an oil bath on a hot plate until boiled. *4.04* *mL* of a *10%* sodium citrate solution (*0.39M* sodium citrate tribasic dihydrate *98%*, Sigma cas 6132-04-3) was then quickly added. The solution was stirred for *5* minutes, and then the flask was removed from the hot oil and placed aside until cooled. The final concentration of the GNPs reaches *30* *mg/ml*.

Uncoated spherical GNPs tend to aggregate because of their negative charge (due to the citric acid stabilizing ligand). One way to prevent aggregation it is to coat them with protein^36^. Another method we used in order to prevent aggregation and stabilize the particles in physiological solutions, *100* *μL O*-(2-Carboxyethyl)-*O*′-(2-mercaptoethyl)heptaethylene glycol (PEG7) (*95%*, MW *458.56g/mol*, Sigma-Aldrich, Israel Ltd.) was absorbed onto the nanospheres. This layer also provides the chemical groups required for antibody conjugation (-COOH). First, the solution was centrifuged to dispose of excess citrate. PEG7 solution was then added to the solution, stirred at room temperature overnight and put in a centrifuge in order to dispose of excess PEG7.

In order to increase cell-uptake rate, stabilized nanospheres were further coated with glucose. Excess of *100* *μL* EDC (N-ethyl-N -(3-dimethylaminopropyl) carbodiimide) and *100μL* NHS (N-hydroxysuccinimide) (Thermo Fisher Scientific, Inc, Rockford, IL) were added to the solution, followed by addition of *200* *μL* Glucose-2 (2GF)(D-(+)-Glucosamine hydrochloride, Sigma-Aldrich, Israel Ltd.). NHS and EDC form an active ester intermediate with the -COOH functional groups, which can then undergo an amidation reaction with the glucose –NH_2_ group. The solution was stirred at room temperature for *3* hours and put in a centrifuge in order to dispose of excess materials. This protective layer prevents aggregation of the spherical GNPs within the biological sample^37^.

### Synthesis and conjugation of rods GNPs

Rods GNPs (shape *15X55* *nm*, peak wavelength at *785* *nm*) were synthesized using the seed mediated growth method^38^. This method, charges the rods GNPs with a positive charge, which prevent their aggregation within a biological sample. A solution of GNRs suspended in cetyltrimethylammonium bromide (CTAB) (Sigma-Aldrich, USA) was centrifuged at *11 000* *g* for *10* minutes, decanted and resuspended in water to remove excess CTAB. In order to stabilize the particles in physiological solutions, a layer of polyethylene glycol (mPEG-SH, MW *5000* *g/mol*) (creative PEGWorks, Winston Salem, USA) was adsorbed onto the GNRs. This layer also provided the chemical groups that are required for antibody conjugation (SH–PEG–COOH, MW *3400g/mol*). A *200* *ml* mixture of mPEG–SH (*5mM*) (*85%*) and SH–PEG–COOH (*1mM*) (*15%*) was added to *1* *ml* of GNR solution. The mixture was stirred for *24* hours at room temperature. The absorption spectrum of PEGylated GNR solution presented the same absorption peak at *785* *nm*. The heterofunctional PEG was covalently conjugated to a CC49 monoclonal antibody, which is specific to the TAG-72 antigen^39^.

### Cell uploading with spherical and rods GNPs

A431 cells were cultured in glucose-free DMEM medium containing *5%* FCS, *0.5%* penicillin and *0.5%* glutamine. Cells were centrifuged and a saline solution at concentration of 0.9% containing nanospheres was added in excess. The cells were then incubated at *37* *ºC* for *1.5* hour. After incubation, the cells were centrifuged twice (*7*  minutes in *1000* rpm) to wash out unbound nanospheres. Then, cells were incubated at 37 °C for *15* minutes with a saline solution containing gold nanorods. After incubation, the cells were centrifuged twice (*7*  minutes in *1000* rpm) to wash out unbound rods.

## Experimental results

**Figure 2:**
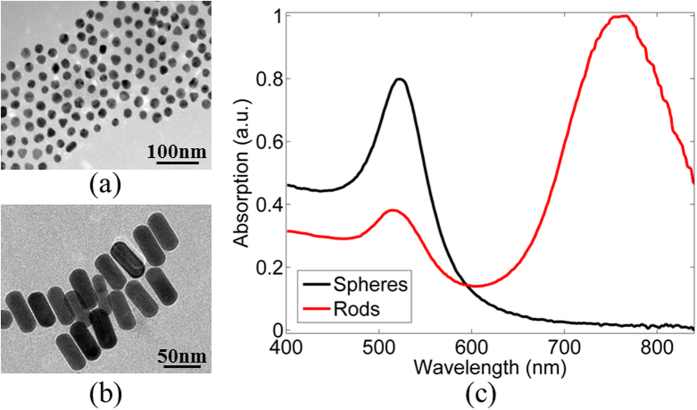
Characterization of GNPs. (**a**) TEM image of spheres GNPs. (**b**) TEM image of rods GNPs. (**c**) Absorption spectrum of the GNPs.

Samples of human epidermoid carcinoma cell line, A431^40^, were injected with *20* *nm* spheres GNPs and *15* *nm x 50* *nm* rods GNPs immobilized on a coverslip, using a known protocol^32^,^41^ (the sample preparation is described in the methods section). Particles characteristics were measured using transmission electron microscopy (TEM) where the spheres diameter was verified to be 20 *nm* (Fig. 2(a)), the rods dimensions were verified to be *15* *nm* × 50 *nm* (Fig. 2(b)). Their absorption spectrum was measured (using the NanoDrop2000c by Thermo-Scientific) and is shown in Fig. 2(c). The spheres are marked in black, where a clear peak at *532* *nm* is presented. The rods GNPs are marked in red and have two peaks; the dominant is at *785* *nm* that is stronger by a factor of *2.5* than the minor peak that is located at *532* *nm*. This minor peak is 2 times weaker than the same peak of the spheres GNPs. The scattering peak of the GNPs was also measured using a visible spectrophotometer (Cary 5000 by Agilent) and demonstrated the same ratios between the peaks as presented in Fig. 2(c).

**Figure 3:**
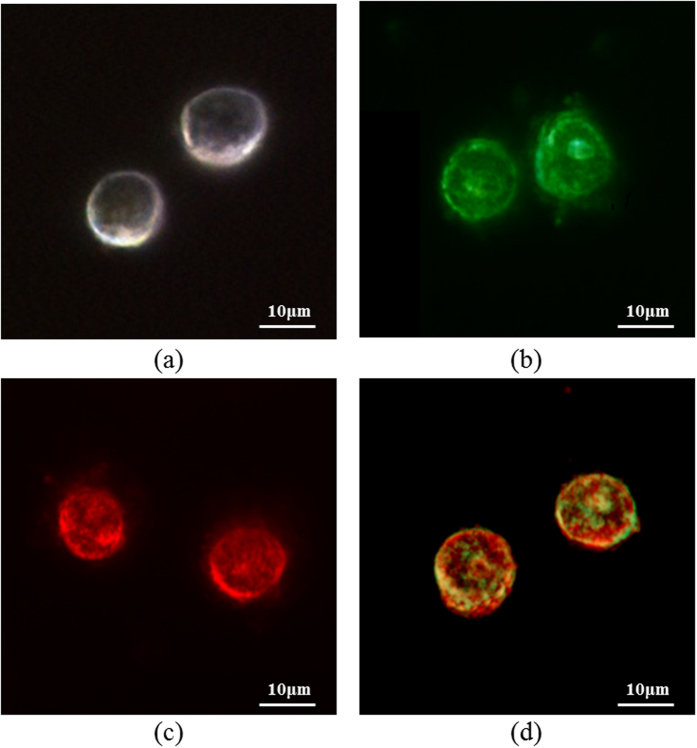
Dark field image of 4 different samples. (**a**) Cells only. (**b**) Cells tagged with spherical GNPs. (**c**) Cells tagged with rods GNPs. (d) Cells tagged with both spherical and rods GNPs.

The GNPs weren’t targeted into a specific area within a cell and therefore they are randomly distributed inside the cells^42^. In order to visualize the effect of different types of GNPs on the scattering of the cells, 4 different samples were imaged using a dark-field microscope (Nikon i50). The first sample was a control set with cells only (Fig. 3(a)). The second sample was cells tagged with spherical GNPs. Due to their scattering peak they make the cell appear green (Fig. 3(b)). The third sample was cells tagged with rods GNPs. Here, due to their dominant scattering peak they make the cell appear red (Fig. 3(c)). The last sample was of cells that were tagged with both spherical and rods. Therefore, the cells appear to contain both green and red (Fig. 3(d)).

**Figure 4:**
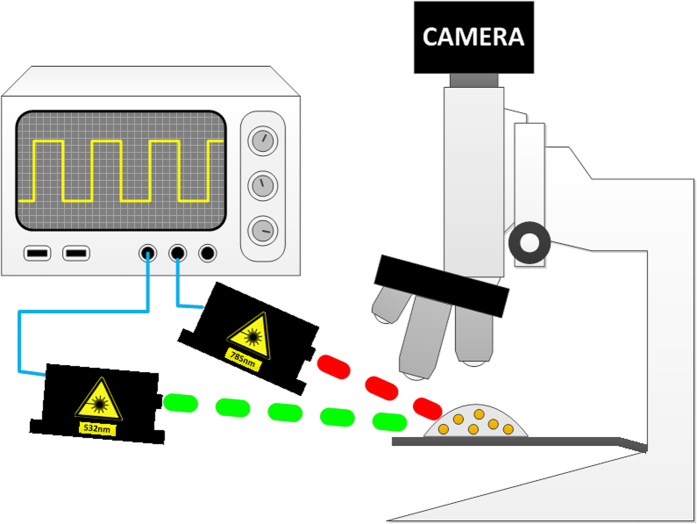
The experimental setup is made of a function generator with two outputs, that modulate a green laser at wavelength of *532* *nm* and a red laser at wavelength of *785* *nm* at two frequencies of *ν^01^* *=* *13* *Hz* and *ν^02^* *=* *25* *Hz* respectively. The modulated beams illuminate the sample and the scattered light is recorded as function of time using a CMOS camera.

The proposed method was tested using the experimental setup that is described in Fig. 4.

**Figure 5:**
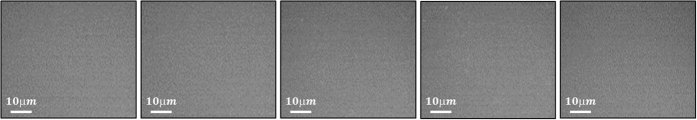
A sequence of recorded images of light scattered from the sample having -27 dB SNR. The irradiation was generated using two laser beams (at wavelengths of *532* *nm* and at *785* *nm*) with modulation frequencies of *13* *Hz* and *25* *Hz* respectively.

**Figure 6:**
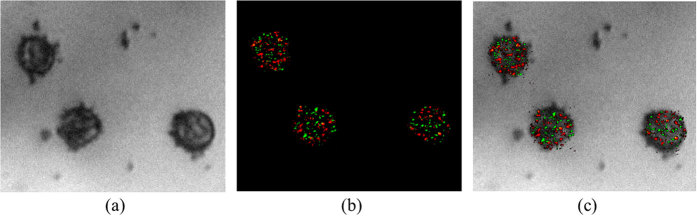
Expeirmentally extracted images. (**a**) A bright field image of the sample. (**b**) The reconstructed image of the sample using TSL for each of the two frequencies (marked in green and red). (**c**) The superimposing of (**a**) and (**b**).

A function generator (AFG3022B by Tektronix) was used to create two square waves with known frequencies of *ν*_*01*_ *=* *13* *Hz and ν*_*02*_ *=* *25* *Hz* (that fulfil the Nyquist sampling criteria as the frame rate of the camera was *215 frames/s*) and a duty cycle of *50%*. The first signal at *ν*_*01*_was connected to the modulation port of a green laser at *532* *nm* (Photop DPGL-2100F) and the second signal at *ν*_*02*_ was connected to the modulation port of a red laser at *785* *nm* (Oxxius LBX-785S). The modulated beams illuminated the sample and the scattered light as a function of time was recorded using a CMOS camera (PixeLink PL-A741-E). The images were taken with parameters of the highest gain of the camera (17.7 dB), low exposure time (*10* *ms*) and low laser powers (6 mW) to mimic high background and significant shot noise conditions. The SNR of the set of images was calculated to be -27 dB. Figure 5 presents a sequence of recorded images of the scattered light from the sample with -27 dB SNR where the GNPs are indistinguishable from the noise. A bright field image of the sample at a size of *750 × 650* pixels was taken with the Olympus BX51 microscope using X*40* objective lens (Fig. 6(a)). The TSL technique was applied to the sequence of recorded images first with the frequency *ν*_*01*_, followed with the same processing at *ν*_*02*_. Since the rods GNPs also have a minor scattering peak at *532* *nm*, the same as that of the spheres, the processed image with *ν*_*01*_ contains both the spherical and the rods GNPs. Therefore, the processed image with *ν*_*02*_, that contains only the rods GNPs, was subtracted from the *ν*_*01*_ image and the result was two images, one for each type of GNP. The final image that represents the two types of GNPs in the sample is the sum of the two images, where for visualization, each type was colored differently, in green and red (Fig. 6(b)). Figure 6(c) is the superimposing of Fig. 6(a,b). The overlap between the locations of the GNPs to that of the cells, indicates that the GNPs are concentrated inside the cells, and by using the ability to attach the GNPs to a specific area within a cell, the proposed technique provides a tool to study intra-cellular processes.

**Figure 7:**
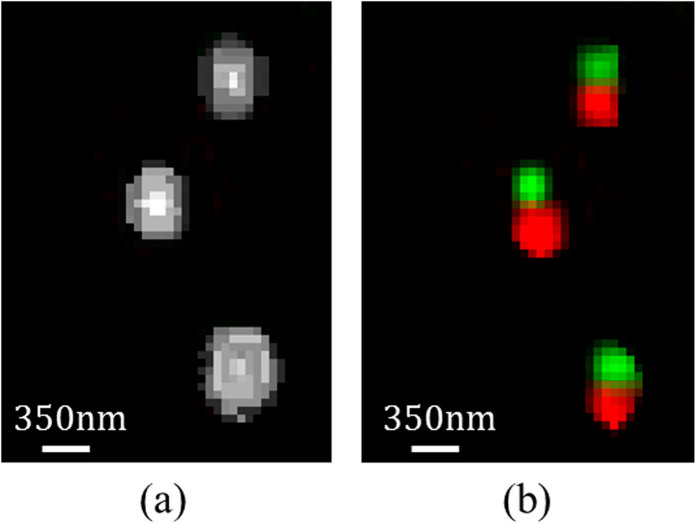
Experimental results. (**a**) Zoom-in on an area inside the sample that contains three spots. The image was captured under conditions of continuous illumination of the sample with the two lasers at high power of 50 mW. (**b**) The same area with the proposed method, where each of the spots contains two different types of GNPs.

In order to validate the ability of the method to detect overlapping GNPs, a reference image of the sample was taken using continuous illumination with the two lasers at high power of 50 mW. The obtained image was of all the GNPs in the sample, where areas of overlapping GNPs appear as larger spots. Figure 7(a) is a zoom-in on a *40 × 50* pixels area inside the sample that contains three spots. The same area with the proposed method is presented in Fig. 7(b), where each of the spots contain two different types of GNPs. The experimental results validated the proposed concept.

## Discussion and conclusions

The TSL technique is an alternative approach for imaging a sample that is labeled with GNPs that provides a tool intra-cellular processes study. It allows the simultaneous detection of multiple types of GNPs within a biological samples or cells and provides high noise immunity which makes it ideal for biological applications. There are various applications for the proposed technique. One is the detection of clustered GNPs, which results in a high resolution image of the sample. In addition, by targeting each type of GNPs to different areas within biological samples or cells, the cite-specified areas within the sample can be simultaneously imaged. Another application can be used in a reported technique that detects multiple types of GNPs within a sample and performs multicolored nanometer-resolution mapping of single proteins^43^. This technique requires the use of a multispectral imaging system, whereas applying our proposed technique, requires only a simple camera, rather than a complex system. The TSL method is generic and can be applied to variety of cells and different types of GNPs, given the appropriate laser's wavelength.

## Additional Information

**How to cite this article**: Ilovitsh, T. *et al.* Cellular superresolved imaging of multiple markers using temporally flickering nanoparticles. *Sci. Rep.* **5**, 10965; doi: 10.1038/srep10965 (2015).
